# Broad Host Tropism of Flaviviruses during the Entry Stage

**DOI:** 10.1128/spectrum.05281-22

**Published:** 2023-03-21

**Authors:** Yanan Zhang, Yiran Yan, Suhua Li, Fei Yuan, Dan Wen, Na Jia, Tao Xiong, Xing Zhang, Aihua Zheng

**Affiliations:** a State Key Laboratory of Integrated Management of Pest Insects and Rodents, Institute of Zoology, Chinese Academy of Sciences, Chaoyang District, Beijing, China; b CAS Center for Excellence in Biotic Interactions, University of Chinese Academy of Sciences, Shijingshan District, Beijing, China; c State Key Laboratory of Pathogen and Biosecurity, Beijing Institute of Microbiology and Epidemiology, Beijing, China; Thomas Jefferson Univeristy

**Keywords:** entry independent, flaviviruses, host tropism, Zika virus

## Abstract

The genus *Flavivirus* consists of viruses with various hosts, including insect-specific flaviviruses (ISFs), mosquito-borne flaviviruses (MBFs), tick-borne flaviviruses (TBFs), and no-known vector (NKV) flaviviruses. Using the reporter viral particle (RVP) system, we found the efficient entry of ISFs into vertebrate cells, MBFs into tick cells, as well as NKVs and TBFs into mosquito cells with similar entry characteristics. By construction of reverse genetics, we found that Yokose virus (YOKV), an NKV, could enter and replicate in mosquito cells but failed to produce infectious particles. The complete removal of the glycosylation modification on the envelope proteins of flaviviruses had no obvious effect on the entry of all MBFs and TBFs. Our results demonstrate an entry-independent host-tropism mechanism and provide a new insight into the evolution of flaviviruses.

**IMPORTANCE** Vector-borne flaviviruses, such as Zika virus, have extremely broad host and cell tropism, even though no critical entry receptors have yet been identified. Using an RVP system, we found the efficient entry of ISFs, MBFs, TBFs, and NKVs into their nonhost cells with similar characteristics. However, glycan-binding proteins cannot serve as universal entry receptors. Our results demonstrate an entry-independent host-tropism mechanism and give a new insight into the cross-species evolution of flaviviruses.

## INTRODUCTION

**FIG 1:**
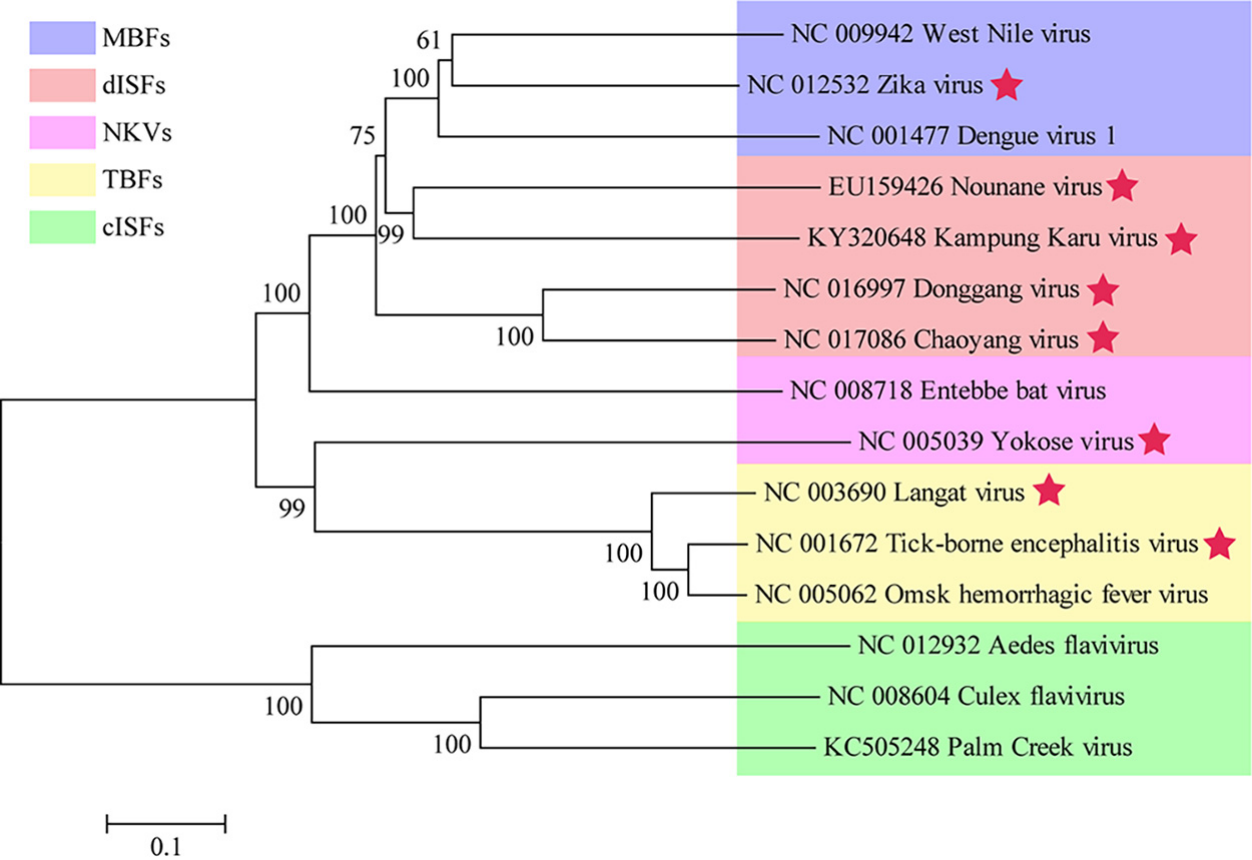
Maximum-likelihood phylogenetic tree of different groups of flaviviruses. Complete polyprotein amino acid sequences were aligned, and a maximum likelihood phylogenetic tree was reconstructed in MEGA7 (41). The consensus tree inferred based on 500 replicates (42) was taken to represent the evolutionary history of the taxa. Stars indicate viruses tested in Fig. 2 and 4. Purple, mosquito-borne flaviviruses (MBFs); orange, dual-host affiliated insect-specific flaviviruses (dISFs); pink, no known vector flaviviruses (NKVs); yellow, tick-borne flaviviruses (TBFs); and green, classical insect-specific flaviviruses (cISFs).

The genus *Flavivirus* contains arthropod-borne flaviviruses, insect-specific flaviviruses (ISFs) (1), and vertebrate-specific flaviviruses (also known as no-known vector flaviviruses [NKVs]) (2). All the known pathogenic flaviviruses are arthropod-borne and are mainly transmitted by mosquitoes and ticks (3). For example, Zika virus (ZIKV), dengue virus (DENV), West Nile virus (WNV), and Japanese Encephalitis virus (JEV) are mosquito-borne flaviviruses (MBFs), while tick-borne encephalitis virus (TBEV) and Langat virus (LGTV) are tick-borne flaviviruses (TBFs). There are also some flaviviruses maintained in arthropod- or vertebrate-restricted transmission cycles. ISFs are isolated only from mosquitos and can be further divided into classical insect-specific flaviviruses (cISFs), such as Niénokoué virus (NIEV) and dual host affiliated, and insect-specific flaviviruses (dISFs), such as Donggang virus (DONV) and Chaoyang virus (CHAOV) (1, 4). NKV flaviviruses, such as Entebbe bat virus and Yokose virus (YOKV), are maintained by vertebrate-only transmission, and most are isolated from bats and rodents (2). Phylogenetic analysis suggests that the flaviviruses can be divided into three evolutionary branches: the first branch includes MBFs and dISFs, NKVs and TBFs are grouped in the second branch, and cISFs are the third branch (Fig. 1). The evolutionary relationship between ISFs, NKVs, and arthropod-borne flaviviruses is still unknown.

Flaviviruses are positive-strand RNA viruses, approximately 11 kb in size, including an approximate 100 nucleotide (nt) 5′ untranslated region (5′ UTR), a single open reading frame (ORF), and approximately 400 to 600 nt 3′ untranslated region (3′ UTR). The ORF encodes three structural proteins, namely, capsid (C), precursor M (prM), and envelope (E) proteins, together with seven nonstructural proteins (5). The prM and E proteins form (prM/E)_3_ trimers and incorporate them onto the immature virus particles in the ER. The precursor M is cleaved into pr+M by a furin protease in the trans-Golgi network, and the resulting mature virus is covered by (M+E)_2_ dimers (6–9). The prM cleavage is not efficient under native conditions and can be fully matured *in vitro* by overexpression of furin (10, 11). The E protein is responsible for the receptor-binding and membrane fusion, which is also the major target of neutralizing antibodies. Virus particles dock on the cell surface by binding with attachment factors, such as heparin sulfate, DC-SIGN, TIM/TAM family, and unknown receptors (12–14). Then, the virus particles are taken up into the cell by clathrin-dependent endocytosis into endosomes, where the low pH triggers membrane fusion (15, 16). Most arthropod-borne flaviviruses have two N-glycosylation sites, one on the pr protein and the other on the E protein, which are involved in protein folding, virus entry, pathogenesis, and host defense (17, 18).

Most viruses have a relatively narrow host range and only replicate in a subset of cell types, such as coronaviruses and adenoviruses. A certain degree of evolution must be achieved when these viruses change or expand their host range. To infect a new host, the virus has to efficiently cross many barriers, such as receptor binding, membrane fusion, protein expression, virus assembly, secretion, and immune defense, among which the initial receptor binding on the cell surface is a critical step in determining the host specificity (19). Studies on the Ebola virus, severe acute respiratory syndrome coronavirus (SARS-CoV), and avian influenza suggest that the emergence of critical mutations plays an important role in the cross-species transmission of these viruses (20). Although heparin sulfate, DC-SIGN, and TIM/TAM family are involved in the entry of flaviviruses (12–14), the receptor usage of flaviviruses is largely unknown, which limits the study of the cross-species transmission of flaviviruses.

MBFs and TBFs could transmit among vertebrate hosts through mosquitoes or tick bites (21, 22). However, ISFs or NKVs are maintained solely in arthropods or vertebrates (2, 23). Here, we applied pseudoinfectious reporter virus particles (RVPs) (24), an entry-studying tool, to study the cross-species entry of flaviviruses. Collectively, we found a universal cross-species transmission mechanism of flaviviruses that is different from that in other virus groups.

## RESULTS

### RVPs of dISFs, cISFs, and NKV can infect human and mosquito cells.

**FIG 2:**
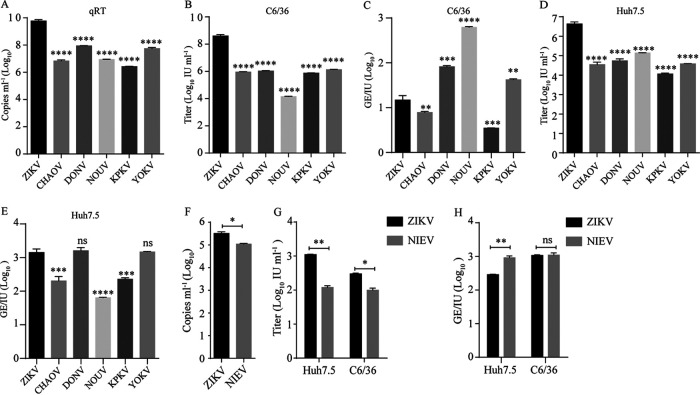
RVPs of ISF and NKV can infect mosquito and human cells. (A to E) RVPs were produced by cotransfection of CprME-expression plasmid and WNV replicon encoding a GFP reporter in 293T cells. (A) RVP secretion was measured by real-time PCR with primers according to the WNV replicon. RVP titers in the supernatant were gauged on C6/36 (B) or Huh7.5 (D) cells 72 h post-transfection. (C and E) The infectivity of RVPs was further calculated as GE/IU. The data were analyzed by unpaired *t* *t*est (*n* = 3). (F to H) cISF RVP was produced by cotransfection in C6/36 cells. (F) cISF RVP secretion was measured by real-time PCR with primers based on the WNV replicon. (G) RVP titer was gauged on Huh7.5 and C6/36 cells. (H) The infectivity of cISF RVP is shown as GE/IU. The data were analyzed by two-tailed unpaired *t* test (*n* = 3). Error bars indicate standard deviation (SD). The results show the average and standard deviation of three independent experiments; ns, not significant, *P ≥ *0.05; *, *P* < 0.05; **, *P* < 0.01, ***; *P* < 0.001; ****, *P < *0.0001.

Previously, we found that while dISFs could not infect vertebrates, they could enter vertebrate cells efficiently but were unable to initiate replication (23). Thus, we wondered whether NKV flaviviruses could enter mosquito cells. To test this hypothesis, we applied an RVP system by which the exogenous C-prM-E structural proteins were incorporated into the WNV replicon encoding a green fluorescent protein (GFP) reporter (24). The RVPs only support single-round infection, and the WNV replicon can replicate in both vertebrate and mosquito cells. YOKV (Oita-36 strain) is an NKV flavivirus and was isolated from a bat in Japan in 1971. RVPs of YOKV, as well as four dISFs (DONV, CHOAV, Nounané virus [NOUV], and Kampung Karu virus [KPKV]) and ZIKV, were prepared by cotransfection of C-prM-E expressing plasmids and the WNV replicon plasmid into 293T cells as previously described (24, 25). The secretion of RVPs was determined using real-time PCR by detecting the copies of the WNV replicon in the supernatants. As shown in Fig. 2A, the RVPs of YOKV, as well as dISFs, were successfully secreted despite their RNA copy numbers being about 2 logs lower than those of ZIKV (Fig. 2A). The titers of RVPs in the supernatant were measured in both the mosquito cell line C6/36 and human cell line Huh7.5 by counting the GFP-positive cells. The RVPs of YOKV, as well as dISFs, were able to infect both Huh7.5 and C6/36, exhibiting lower titers than ZIKV as measured by infectious unit (IU) per milliliter of supernatant (Fig. 2B and D). The infectivity of RVPs was expressed as genome (GE)/IU as described before (23). The infectivity of YOKV in C6/36 cells and dISFs in Huh7.5 cells was comparable to those of ZIKV (Fig. 2C and E). These results suggested that YOKV can enter mosquito cells efficiently and entry is not a barrier for the cross-species transmission of flaviviruses.

We failed to assemble the RVPs of cISFs in 293T cells, probably due to the distant phylogenetic relation to the other flaviviruses. Thus, we tried to assemble the RVPs in mosquito cells. NIEV is a cISF isolated from *Culex* mosquitoes in Côte d’Ivoire (26). The NIEV RVP was successfully packaged in C6/36 cells (Fig. 2F). NIEV RVP could infect human cells, as well as mosquito cells, with lower titers than ZIKV (Fig. 2G). The infectivity of NIEV in Huh7.5 cells was comparable to that of ZIKV (Fig. 2H). These results indicated that cISFs could enter vertebrate cells as dISFs.

### Cross-species barrier of YOKV occurs post-entry.

**FIG 3:**
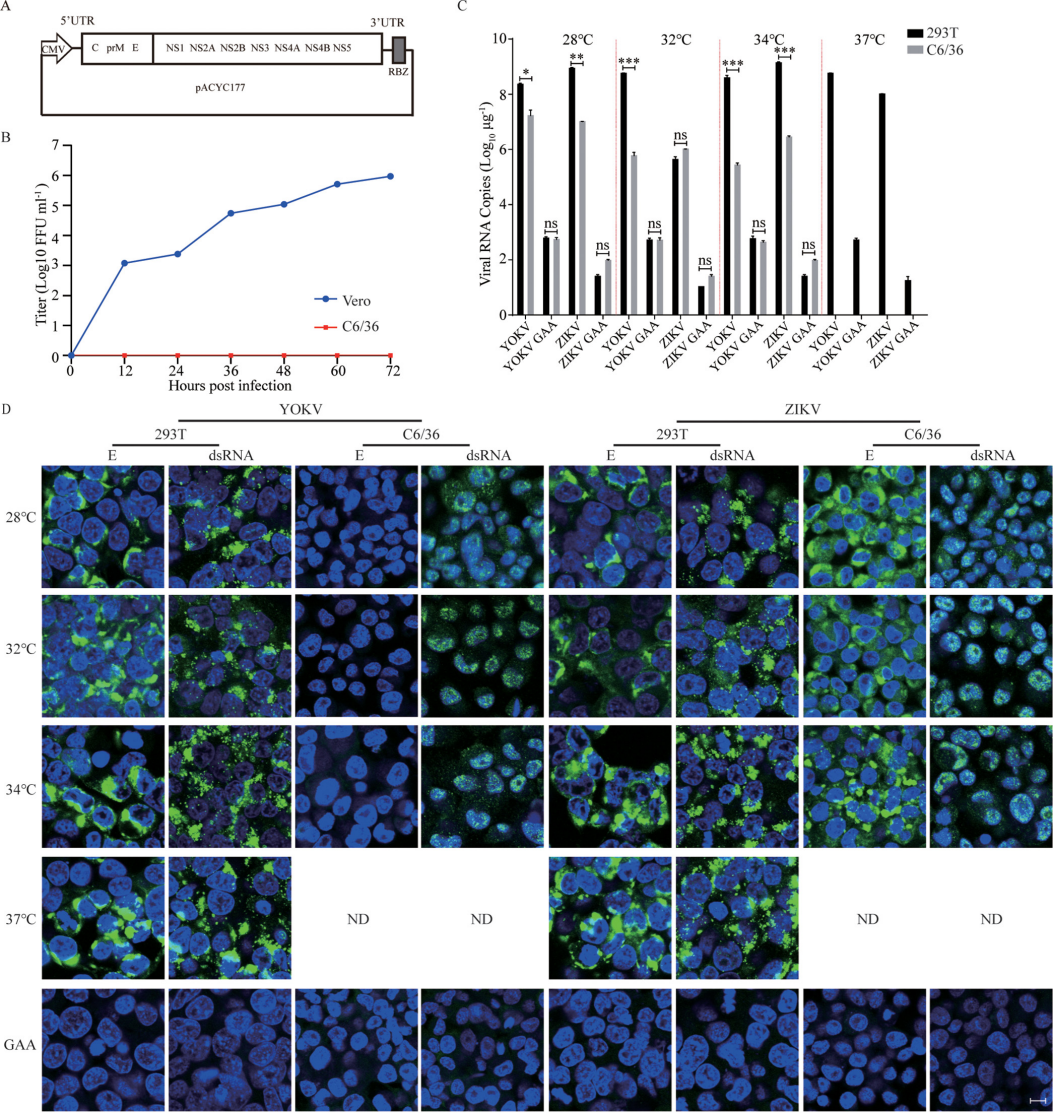
YOKV can replicate in mosquito cells. (A) Schematic diagram of the YOKV infectious clone. The cDNA of YOKV was cloned into the pACYC177 vector with a CMV promoter and HDV RBZ termination sequence. (B) Viral titers in the supernatant were determined at the indicated time points by a focus-forming assay. YOKV titers peaked at 72 h post-infection at around 10^6^ FFU/mL (*n* = 3). (C) Virus replication was detected by real-time PCR in transfected cells at indicated temperatures. The replication-deficient GAA mutations were negative controls (*n* = 3). Error bars indicate standard deviation (SD). (D) Immunofluorescence of viral double-stranded RNA by anti-dsRNA mAb and envelop protein E by 4G2 mAb in transfected vertebrate cells and insect cells under various temperatures. ND, not detected. The nuclei were stained with Hoechst 33342; scale bar, 10 μm. GAA is a replication-deficient mutation. These results show the average and standard deviation of three independent experiments. The data were analyzed by two-tailed multiple *t* test; ns, not significant, *P ≥ *0.05; *, *P < *0.05; **, *P < *0.01; ***, *P < *0.001; ****, *P < *0.0001.

To further characterize the cross-species barrier of NKV in mosquito cells, we constructed an infectious clone of YOKV using the same strategy as ZIKV Natal-RGN (27). Viral RNA transcription was driven by a mammalian CMV promoter, which also works in insect cells (28) (Fig. 3A). We successfully rescued the virus and the infectious YOKV propagated efficiently in Vero cells, with a titer of 10^6^ focus-forming units (FFU)/mL at 72 h after infection, while no propagation was detected in C6/36 cells (Fig. 3B). We transfected the plasmid of YOKV and ZIKV infectious clones under a CMV promoter into mosquito cells C6/36 and vertebrate cells 293T and cultured at different temperatures ranging from 28°C to 37°C. Efficient replication of YOKV was detected in 293T from 28°C to 37°C and in C6/36 from 28°C to 34°C with the exception of 37°C due to cell death. Robust YOKV E protein and double-strand RNA (dsRNA) was observed in 293T cells by immunofluorescence post-transfection. In contrast, only dsRNAs of YOKV were detected in C6/36 cells (Fig. 3C and D). It is plausible that the E protein is not properly processed or has reduced stability in mosquito cells.

**FIG 4:**
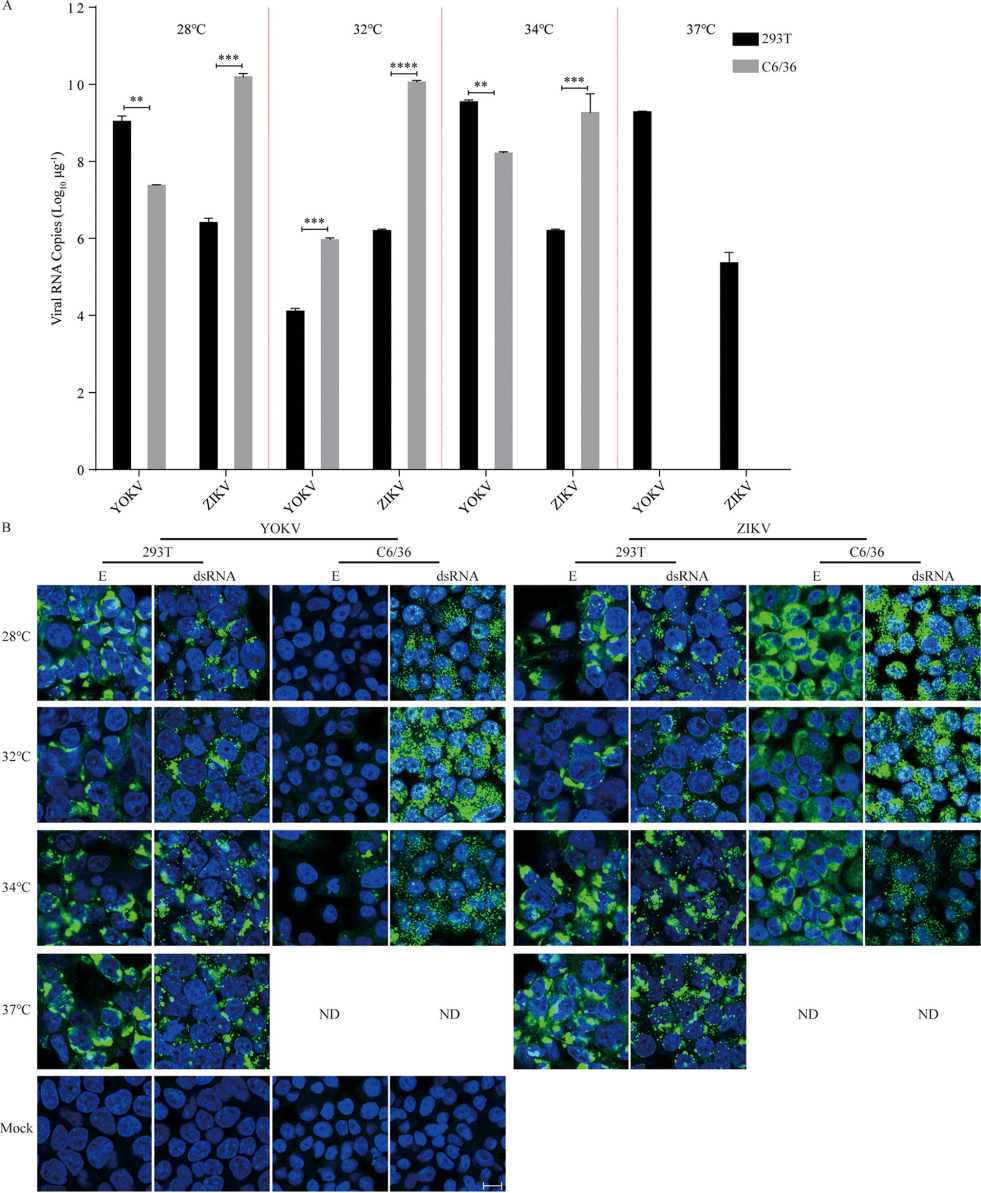
Cross-species barrier of YOKV occurs post-entry. (A) Virus replication was detected by real-time PCR in infected cells at different temperatures. Cells were infected at an MOI of 0.5 and cultured at the indicated temperatures. Viral RNAs were tested by real-time PCR 72 h postinfection. Error bars indicate standard deviation (SD). (B) Immunofluorescence of viral double-stranded RNA by anti-dsRNA mAb and envelop protein E by 4G2 mAb in infected vertebrate cells and insect cells under indicated temperatures. ND, not detection. The nuclei were stained with Hoechst 33342; scale bar, 10 μm; *n* = 3. These results show the average and standard deviation of three independent experiments. The data were analyzed by two-tailed multiple *t* test; ns, not significant, *P ≥ *0.05; *, *P < *0.05; **, *P < *0.01; ***, *P < *0.001; ****, *P < *0.0001.

To further prove this hypothesis, we infected C6/36 and 293T with YOKV and ZIKV, respectively, at 28°C to 37°C. About 2-log higher of YOKV viral RNA level in C6/36 (Fig. 4A) was observed compared with those by transfection of the infectious clones (Fig. 3C). Correspondingly, a low level of YOKV E protein expression was detected in C6/36 cells at 34°C but not at 28°C and 32°C. However, still no infectious YOKV particles were produced in the C6/36 supernatants at all temperatures (data not shown). These results suggest that YOKV can replicate in mosquito cells but the viral proteins are not properly processed or unstable at the physiological temperature.

### RVPs of TBFs can infect mosquito cells and RVPs of MBFs can infect tick cells as well.

**FIG 5:**
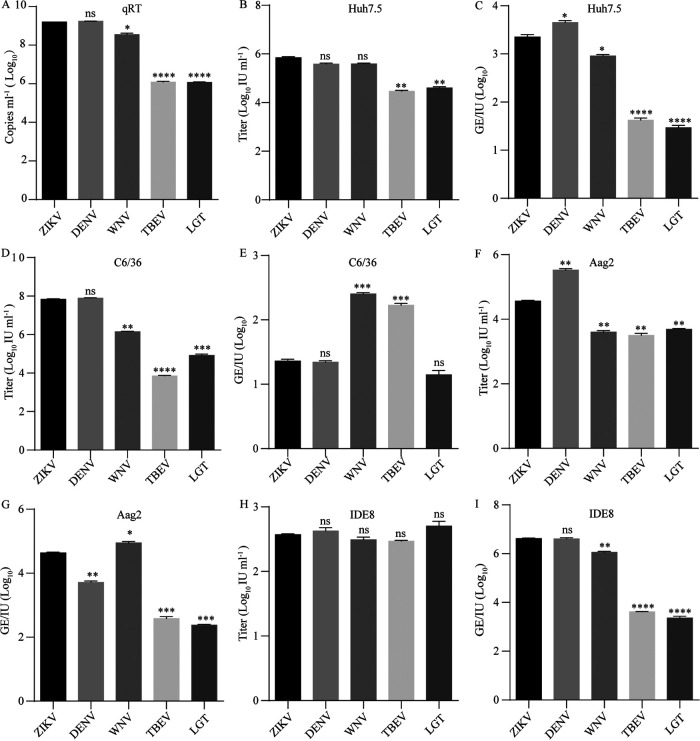
RVPs of TBFs can infect mosquito cells and MBFs are able to infect tick cells. RVPs of TBFs (TBEV and LGTV) and MBFs (ZIKV, DENV, and WNV) were produced as above. (A) RVP secretion was measured by real-time PCR with primers based on the WNV replicon. RVP titers in the supernatant were gauged on Huh7.5 (B), C6/36 (D), Aag2 (F) and IDE8 (H) cells at 72 h posttransfection. (C, E, G, and I) The infectivity of TBFs and MBFs is shown as GE/IU (*n* = 3). Error bars indicate standard deviation (SD). The results show the average and standard deviation of three independent experiments. The data were analyzed by two-tailed unpaired *t* test; ns, not significant, *P ≥ *0.05; *, *P < *0.05; **, *P < *0.01; ***, *P < *0.001; ****, *P < *0.0001.

Phylogenetic analysis suggested that TBFs were clustered in a clade sister to MBFs (Fig. 1). Many studies demonstrated that TBFs, such as TBEV and LGTV, could not infect mosquitos and mosquito cell lines (29–32). To test whether TBFs have barriers to entering mosquito cells, we prepared RVPs of TBEV and LGTV. The secretion of TBEV and LGTV were approximately 2 to 3 logs lower than ZIKV as determined by the RNA genome copies in the supernatant (Fig. 5A). The RVP titers in the supernatants were measured in human Huh7.5 cells and two mosquito cell lines, C6/36 and Aag2. The titers of TBEV in Huh7.5 and C6/36 cells were 1.5 and 0.6 logs lower than those of ZIKV, respectively, while the titers of LGTV in Huh7.5 and C6/36 cells were 2.4 and 1.5 logs lower than those of ZIKV, respectively (Fig. 5B and D). The titers of both TBEV and LGTV in Aag2 cells were slightly higher than those of ZIKV (Fig. 5F). The infectivity of TBEV and LGTV in two mosquito cell lines, expressed as GE/IU, was comparable or even higher than that of ZIKV (Fig. 5E and G). These results indicated that TBFs have no entry barrier in mosquito cells.

Conversely, we also tested the entry of MBFs in tick cells. MBF RVPs, ZIKV, DENV, and WNV, could infect tick cells IDE8 (Fig. 5H), with the titers of MBFs were comparable to TBFs. While the infectivity of MBFs to tick cells, expressed as GE/IU, was lower than TBFs (Fig. 5I). In conclusion, there is also no entry barrier of MBFs in tick cells.

### Flavivirus RVPs are internalized through the same pathway.

**FIG 6:**
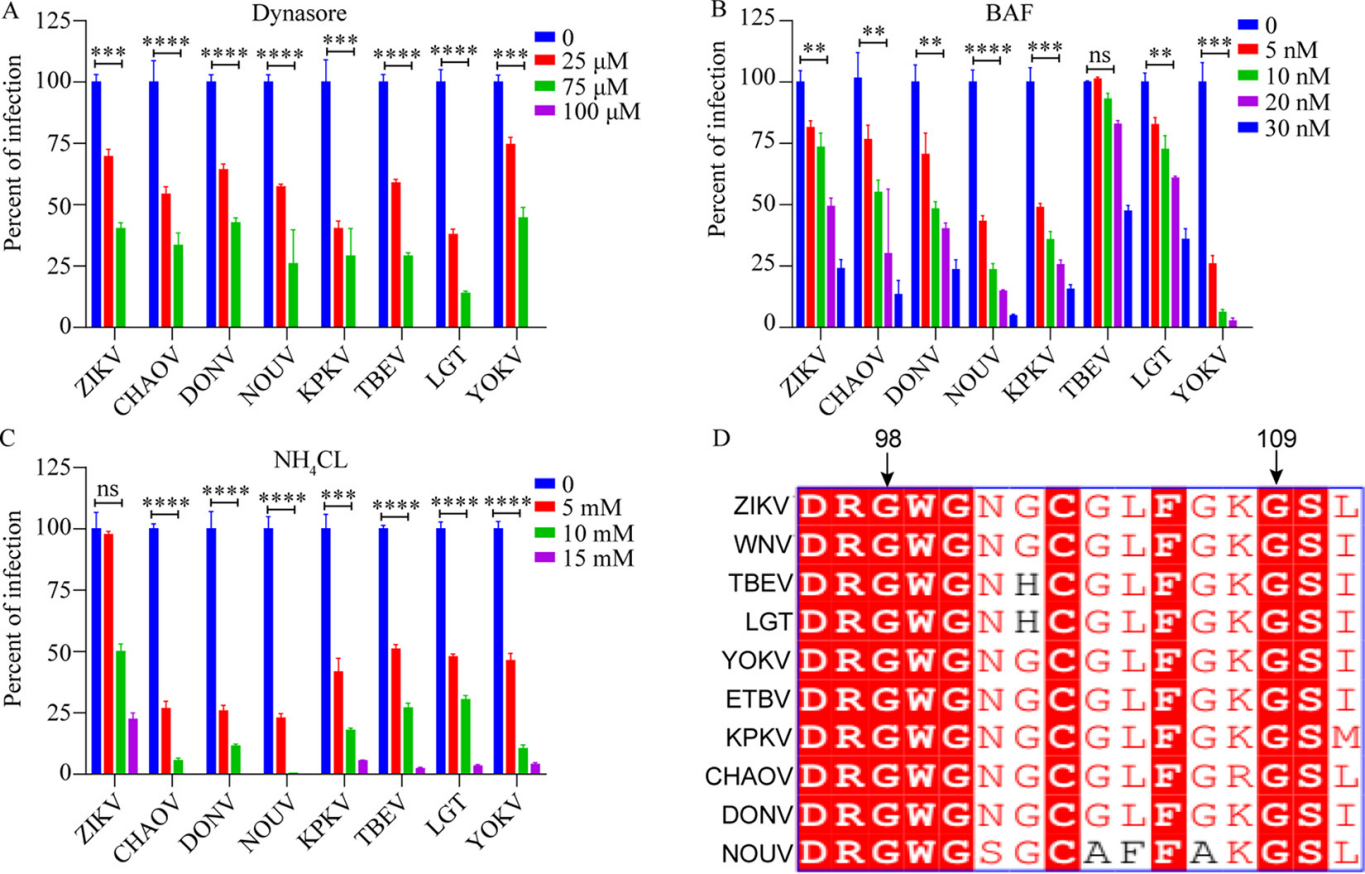
ISFs, NKVs, and MBFs are similar in the endocytosis and membrane fusion. Huh7.5 cells were pretreated with indicated concentrations of dynasore (A), bafilomycin A (BAF) (B), or NH_4_Cl (C). Then, 1 h later, indicated RVPs were added to Huh7.5 cells. The infectivity of all RVPs was inhibited by the compounds in a similar pattern. (D) Alignment of the fusion loop of dISFs, NKVs, TBFs, and MBFs (*n* = 3). Error bars indicate standard deviation (SD). The results show the average and standard deviation of three independent experiments. The data were analyzed by two-tailed multiple *t* test; ns, not significant, *P* ≥ 0.05; **, *P* < 0.01; ***, *P* < 0.001; ****, *P* < 0.0001.

After binding with cellular receptors, arthropod-borne flaviviruses, such as ZIKV and WNV, are internalized through clathrin-dependent endocytosis into the endocytic compartments. Then, the low pH in the late endosome triggers E protein-mediated membrane fusion. As such, we then tested whether all flaviviruses are internalized through the same pathway. Pretreatment of Huh7.5 cells with dynasore, an inhibitor of dynamin critical for clathrin-mediated endocytosis, effectively blocked infection of ZIKV RVPs in a dose-dependent manner. Infection with dISFs, NKV, and TBFs RVPs was reduced in a similar pattern, suggesting that they are all internalized via clathrin-mediated endocytosis (Fig. 6A).

Since ZIKV fusion was low-pH dependent, it was inhibited by the mild alkaline, amino chloride (NH_4_Cl), and bafilomycin A (BAF) (Fig. 6B and C), which neutralized the acidic endosome and inhibited vacuolar-type H^+^-ATPase. To analyze the pH-dependent fusion of dISF, NKV, and TBF infection, we treated Huh7.5 cells with NH_4_Cl or BAF before RVP infection. Both NH_4_Cl and BAF reduced the infection of dISFs (DONV, CHAOV, NOUV, and KPKV), TBFs (TBEV and LGTV), and NKV (YOKV) in a concentration-dependent manner (Fig. 6B and C). These results demonstrated that dISFs, TBFs, and NKV require low pH for cellular entry into human cells. Sequence alignment of E proteins revealed that fusion loops (residues 98 to 109 of ZIKV E) were highly conserved, suggesting a conserved membrane fusion mechanism among MBFs, dISFs, TBFs, and NKVs (Fig. 6D). These results demonstrated that flaviviruses use a similar pathway to enter into cells.

### Glycan modification of the envelope protein is not critical for flavivirus entry.

**FIG 7:**
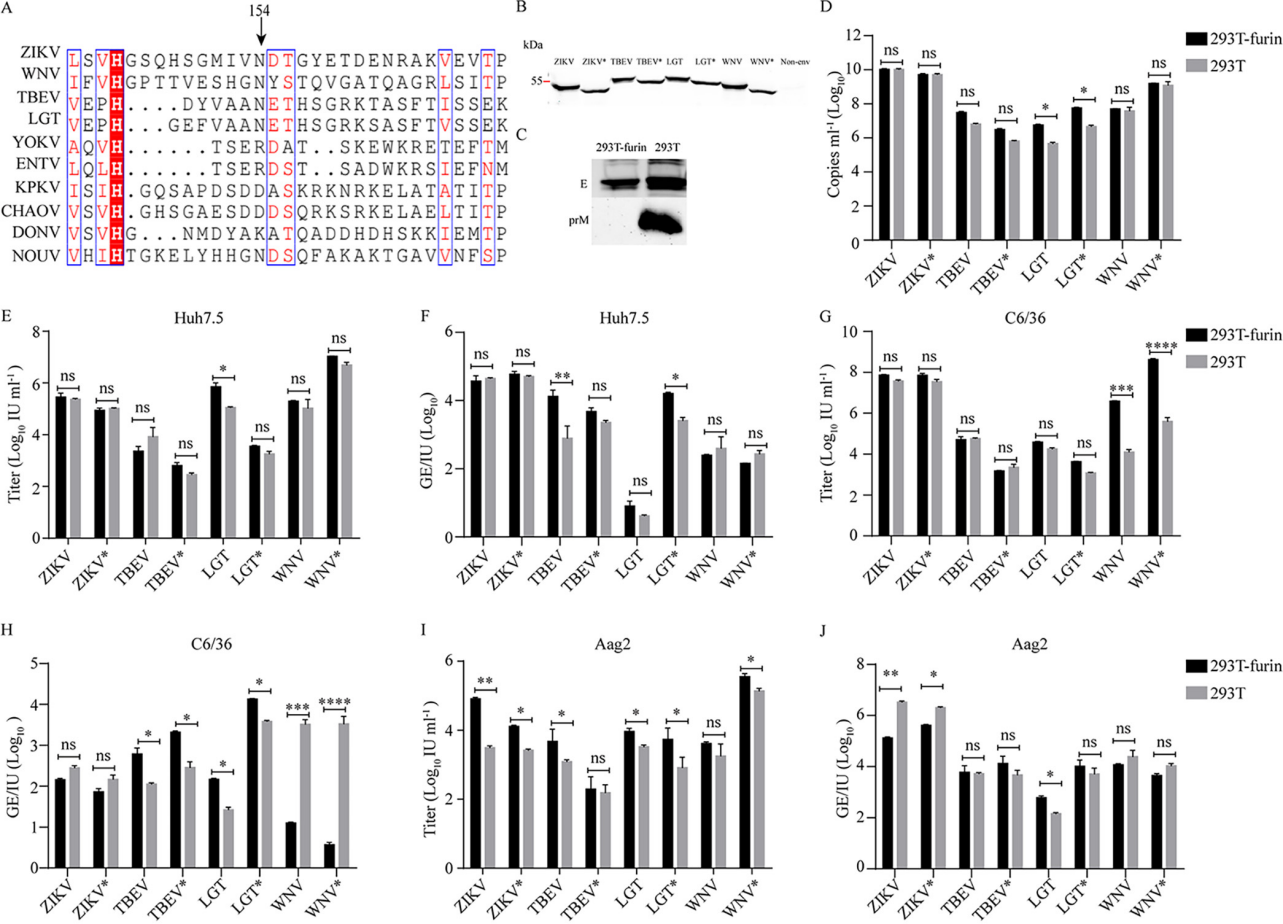
N-glycosylation of flavivirus envelope proteins is not responsible for virus entry. (A) Alignment analysis of the N154 (according to the amino acid sequence of the E protein of ZIKV MR766 NIID strain) glycosylation site of flaviviruses. (B) The removal of the N-glycosylation site was confirmed by Western blot with an anti-E antibody. The mutant migrated faster than the WT. (C) The maturation activity of overexpressed furin was confirmed with a polyclonal antibody against ZIKV prM. prM was detected on ZIKV RVPs produced by naive 293T cells but not by cells with furin overexpression. WT and glycosylation-negative mutant RVPs were produced in naive 293T cells and 293T cells overexpressing a furin protease. RVP titers were gauged on Huh7.5, C6/36, and Aag2 cells. (D) RVP secretion was measured by real-time PCR with primers based on the WNV replicon. (E, G, and I) RVP titers in the supernatant were gauged on Huh7.5 (E), C6/36 (G), and Aag2 (I) cells 72 h post-transfection. (F, H, and J) The infectivity of TBFs and MBFs is shown as GE/IU (*n* = 3). Error bars indicate standard deviation (SD). The results show the average and standard deviation of three independent experiments. The data were analyzed by two-tailed multiple *t* test; ns, not significant, *P ≥ *0.05; *, *P < *0.05; **, *P < *0.01; ***, *P < *0.001; ****, *P < *0.0001.

It was speculated that glycans on the envelope of arthropod-borne flaviviruses might serve as a universal receptor-binding ligand due to the smooth surface structure and broad host tropism. Thus, glycan-binding proteins might serve as universal receptors, resulting in the broad host tropism during the entry stage. Structural studies and glycosylation prediction suggested that all arthropod-borne flaviviruses have a conserved N-glycosylation site on the E protein, except YFV, which has none, and DENV, which has one extra glycosylation site. In contrast, among the ISFs and NKV flaviviruses, only NOUV has one N-glycosylation site on the E protein (Fig. 7A).

To assess the involvement of surface glycans in flavivirus entry, we mutated the N-X-(S/T) motifs of ZIKV, WNV, TBEV, and LGTV E proteins into N-X-I and generated RVPs without N-glycosylation on E proteins (marked with “*”) as described previously (27). Western blot analysis using an anti-E antibody showed that all of the mutant E proteins migrated faster than their wild-type (WT) counterparts, confirming the removal of the N-glycosylation (Fig. 7B). In addition to eliminating the effects of glycosylation on pr proteins from partially matured particles, we produced the complete mature RVPs in 293T cells overexpressing a furin protease (10). The full maturation of ZIKV RVPs from 293T-furin cells was confirmed by Western blot using an anti-ZIKV prM polyclonal antibody. Figure 7C shows that no pr was detected in the ZIKV RVPs from 293T-furin cells compared with samples from naive 293T cells. Removing the N-glycosylation from E or overexpression furin in the packaging cells did not show a significant effect on the secretion of RVPs (Fig. 7D). The RVP titers in the supernatants were tested in the human cell line Huh7.5, Aedes albopictus cell line C6/36, and Aedes aegypti cell line Aag2 (Fig. 7E, G, and I) and the infectivity of WT and mutant RVPs was calculated as above (Fig. 7F, H, and J). After calculation, the infectivity of ZIKV, TBEV, LGT, and WNV in the three cell lines was not significantly affected by the removal of N-glycosylation besides. As expected, 293T-furin cells produced RVPs with no glycan on the viral particles and showed slightly increased infectivity due to full maturation. These results demonstrated that the glycan modification of envelope proteins does not play a key role in the entry process of flaviviruses.

## DISCUSSION

Arthropod-borne flaviviruses can adapt to the distinct cellular environments of arthropods and vertebrates, which belong to different phyla. Since the receptor-virus interaction requires precise structural complementation, receptor adaptation is thus considered to be a critical barrier for cross-species viral transmission. However, we found that ISFs could efficiently enter vertebrate cells and NKVs could efficiently enter mosquito cells, despite their distinct host range. Furthermore, we found that TBFs, which phylogenetically are in a sister clade to MBFs, could enter mosquito cells efficiently. Mutagenesis suggested that flaviviruses do not enter cells by engagement of a universal cellular glycan-binding protein via viral surface glycans. Our results indicated that the cross-species transmission of flaviviruses is unique compared with other virus families.

The dual-host adaption of arthropod-borne flaviviruses needs to cross many infection barriers, of which entry is the first. Here, and in our previous report (23), we applied the RVP system by which the WNV replicon encoding a GFP reporter was pseudotyped with exogenous C-prM-E structural proteins to study flaviviruses entry (24). All the dISFs and NKV we tested, including DONV, CHOAV, NOUV, KPKV, and YOKV, could enter human cells and mosquito cells as efficiently as ZIKV. Furthermore, using an infectious clone, we found that DONV could enter mammalian cells but could not initiate replication (23), NKV could enter mosquito cells, and the cross-species barrier of NKV occurred postentry. TBFs, which have not been isolated from mosquitos and have failed to replicate in mosquitos by experimental infection (29–31), also entered mosquito cells efficiently as revealed by the RVP system. cISFs are phylogenetically located in a distant branch compared with other flaviviruses. A previous study found that NIEV has several infection barriers in vertebrates, including entry using an infectious clone (26). Here, we successfully assembled RVPs of NIEV in C6/36 cells and found NIEV could also enter vertebrate cells efficiently. In contrast to dISF flaviviruses, cISF flavivirus RVP assembly is only achieved in C6/36 cells and NIEV RVPs are able infect both human and mosquito cells, suggesting assembly might be a cross-species barrier of propagation in the vertebrate of cISF. Thus, we speculated that all flaviviruses have no entry barrier in arthropods and vertebrates.

dISFs and NKVs are phylogenetically located in the MBF group with a conserved fusion loop in the E protein, suggesting their close evolutionary relationship. Our results suggested that dISFs and NKV can deliver their genomes into the cytoplasm of vertebrate cells and mosquito cells, respectively, and have the potential to evolve into arboviruses by continuous mutation, which deserves more surveillance and attention. In our previous study, using dISF-MBF chimeras, we found that UTRs of DONV play a key role in the cross-species adaption in vertebrates and critical secondary RNA structures were further mapped (23). Therefore, engineering infectious clones of ISFs and NKVs, together with other MBFs, will uncover the mechanism underlying the dual-host adaptation of MBFs.

We found that all flaviviruses enter cells through receptor-dependent endocytosis and fuse in the endosome at low pH. The receptor usage of arboviruses is complicated. Several cellular receptors of alphaviruses have been identified, such as Mxra8 for arthritogenic alphaviruses, LDLRAD3 for Venezuelan equine encephalitis virus, and VLDLR and ApoER2 for multiple alphaviruses (33–35). However, the knockout of these receptors failed to abolish virus entry, suggesting that extra receptors are under investigation. In addition, the receptor usage of flaviviruses is largely unknown. The relation between host range and receptor conservativity was performed by a comprehensive review of 88 human viruses with at least one receptor, and the results revealed that human viruses with a broad host range tend to use highly conserved proteins as receptors (36). As discussed above, the host range of flaviviruses is extraordinarily broad, crossing both arthropods and vertebrates. Thus, we speculate that the receptor for flaviviruses might be highly conserved.

## MATERIALS AND METHODS

### Cell lines and antibodies.

C6/36, Aag2, 293T, and Vero were originally obtained from ATCC and Huh7.5 cells were obtained from Charles Rice’s lab via MTA. 293T, Vero, and Huh7.5 cells were cultured in Dulbecco's modified Eagle medium (DMEM) media with 10% FBS, 1% penicillin-streptomycin, and 1% l-glutamine at 37°C with 5% CO_2_. C6/36 cells were cultured in RPMI 1640 media supplemented with 10% FBS, 1% penicillin-streptomycin, and 1% l-glutamine at 28°C with 5% CO_2_. Aag2 cells were cultured in Schneider’s *Drosophila* medium (SDM) supplemented with 10% FBS and 1% penicillin-streptomycin at 28°C. The IDE8 cell line was gift from Lesley Bell-Sakyi and the Tick Cell Biobank and maintained using standard procedures as previously (37). The 4G2 antibody is a mouse monoclonal antibody recognizing the fusion loop of flaviviruses (38, 39). The rabbit anti-ZIKV prM polyclonal antibody was a gift from Fei Deng at the Wuhan Institute of Virology, Chinese Academy of Sciences (40).

### Infectious clones.

The Yokose virus infectious clone was synthesized (SYKMGENE Beijing, China) using AB114858.1 as a template and divided into four pieces, namely, A (1 to 2,640), B (2,641 to 5,520), C (5,521 to 8,400), and D (8,401 to 10,990). All four pieces were assembled into a pACYC177 vector with a CMV promoter at the 5′ end and a hepatitis delta virus (HDV) ribozyme (RBZ) terminal site at the 3′ terminal (27). Yokose virus was rescued by transfection into 293T cells by FuGENE 6 (Promega, USA) at indicated temperatures. The virus was collected at 3 days posttransfection and stored at −80°C. The titer was measured in Vero cells by a focus-forming assay as previously described (23) using 4G2 antibodies. The ZIKV virus infectious clone was synthesized (SYKMGENE Beijing, China) using LC002520.1 as a template, constructed, and rescued as previously described (27).

### Transfection assay.

The cells were plated in 24-well plate at a number of 250,000 of C6/36 cells or 200,000 of 293T cells per well. After 24 h, cells were transfected by FuGENE 6 (Promega, USA). The ratio of plasmids and FuGENE 6 is 1:3 (1 μg plasmids, 3 μL Fugene6 per well). Transfection was done as follows: first, medium and FuGENE 6 were mixed and incubated for 5 min; second, 1 μg of plasmids was added to the FuGENE 6 transfection reagent/medium, mixed immediately, and incubated the FuGENE 6 transfection reagent/DNA mixture for 15 min; and third, the FuGENE 6 transfection reagent/DNA mixture was added to each well of a 24-well plate containing 500 μL of cells in growth medium. Sevnety-two hours posttransfection, viral RNA and E protein expression was tested by real-time PCR and immunofluorescence assay.

### Reporter viral particles.

Human codon-optimized sequences encoding the CprME proteins of DONV (GenBank accession number, NC_016997.1), CHAOV (GenBank accession number NC_017086.1), NOUV (GenBank accession number EU159426.2), KPKV (GenBank accession number KY320648.1), ZIKV (GenBank accession number LC002520.1), LGTV (GenBank accession number AF253419.1), TBEV (GenBank accession number JX498940.1), YOKV (GenBank accession number AB114858.1), and WNV (GenBank accession number ABA62343.1) were synthesized by SYKMGENE Beijing and cloned into the pCDNA3.1 vector. The N^153/154^-X-(S/T) of the E protein was mutated to N^153/154^-X-I by site-directed mutagenesis to produce the N-glycosylation-negative mutant RVPs as described previously (27). RVPs were produced by cotransfection of 9 μg of CprME expression plasmid and 3 μg of WNV replicon plasmid pWNVII-Rep-GFPZeo encoding a GFP reporter into 293T cells by calcium phosphate. Then 12 h later, the medium was replaced with DMEM plus 2% fetal bovine serum, and the RVPs were collected at 72 h posttransfection and stored at −80°C. Fully mature RVPs were produced in 293T cells overexpressing the furin protease (27). The titer of RVPs was measured by counting the GFP-positive cells.

To test the secretion of RVPs, the supernatant was spun by an ultracentrifuge at 30, 000 rpm for 2 h through a 20% sucrose cushion. The WNV replicon RNA was isolated from the pellet and cDNA was transcribed using Prime Script RT reagent kit with gDNA Eraser (TaKaRa) and then assessed by real-time PCR. Quantitative PCR was performed using an SYBR PremixEX *Taq* II (RT)-PCR kit (TaKaRa) on a Thermo PIKOREAL 96 real-time PCR system. The sequences of the WNV replicon primers were as follows: sense, 5′-ACCGCTTCGCCACATCACTACACTT-3′; and antisense, 5′-GAACCTGCTGCCAGTCATACCACCC-3′. The following amplification program was used: incubation at 95°C for 10 s, followed by 40 cycles of 95°C for 5 s and 60°C for 20 s. Information collection and melt curve analysis were done following the instrument’s operation manual. RNA copies were calculated by absolute quantitative PCR.

### Immunofluorescence assay.

Virus-infected cells were fixed with 4% paraformaldehyde for 30 min and permeabilized with 0.5% Triton X-100 for 10 min. Subsequently, cells were blocked with 3% bovine serum albumin in phosphate-buffered saline (PBS) for 1 h, and then the cells were incubated with mouse monoclonal antibody (mAb) 4G2 and mAb J2 (Scicons) antibodies at a dilution of 1:400 for 3 h to detect flavivirus E protein and double-stranded RNA, respectively. After being washed with PBS three times, the cells were incubated with Alexa Fluor 488 goat anti-mouse secondary antibody (1:400) for 1 h. Hoechst 33342 was added at 1 μg/mL to stain the nucleus. The resulting fluorescence was detected by confocal microscopy (Zeiss LSM 710; Germany).

### Viral replication assay.

YOKV and ZIKV infectious clone plasmids were transfected into C6/36 or 293T cells by FuGENE 6 (Promega, USA). Seventy-two hours posttransfection, cellular RNAs were isolated by TRIzol reagent (Invitrogen). RNAs were treated for 1 h by DNase I at 37°C and reverse transcribed using a Prime Script RT reagent kit with gDNA Eraser (TaKaRa). Quantitative PCR was performed as mentioned above.

For infection assay, C6/36 cells or 293T cells were infected at an multiplicity of infection (MOI) of 0.5. After 72 h infection, RNAs were isolated, reverse transcribed, and tested by quantitative PCR as above.

### Statistical analysis.

All results are representative of three independent experiments, and error bars indicate standard deviation (SD). Where appropriate, comparisons were analyzed using two-tailed *t* test with a *P* value of <0.05 being considered statistically significant. The corresponding statistical significance are shown in the figure legends.

## References

[1] Blitvich BJ, Firth AE. 2015. Insect-specific flaviviruses: a systematic review of their discovery, host range, mode of transmission, superinfection exclusion potential and genomic organization. Viruses 7:1927–1959. doi:10.3390/v7041927. 25866904 PMC4411683

[2] Blitvich BJ, Firth AE. 2017. A review of flaviviruses that have no known arthropod vector. Viruses 9:v9060154. doi:10.3390/v9060154. PMC5490829 28635667

[3] Gould EA, Solomon T. 2008. Pathogenic flaviviruses. Lancet 371:500–509. doi:10.1016/S0140-6736(08)60238-X. 18262042

[4] Guzman H, Contreras-Gutierrez MA, Travassos da Rosa APA, Nunes MRT, Cardoso JF, Popov VL, Young KI, Savit C, Wood TG, Widen SG, Watts DM, Hanley KA, Perera D, Fish D, Vasilakis N, Tesh RB. 2018. Characterization of three new insect-specific flaviviruses: their relationship to the mosquito-borne flavivirus pathogens. Am J Trop Med Hyg 98:410–419. doi:10.4269/ajtmh.17-0350. 29016330 PMC5929187

[5] Lindenbach BD, Thiel H-J, Rice CM. 2007. Flaviviruses: the viruses and their replication, p 1101–1152. *In* Knipe DM, Howley PM (ed), Fields' virologv, 5th ed. Lippincott, Williams and Wilkins, Philadelphia, PA.

[6] Lorenz IC, Allison SL, Heinz FX, Helenius A. 2002. Folding and dimerization of tick-borne encephalitis virus envelope proteins prM and E in the endoplasmic reticulum. J Virol 76:5480–5491. doi:10.1128/JVI.76.11.5480-5491.2002. 11991976 PMC137023

[7] Zhang Y, Corver J, Chipman PR, Zhang W, Pletnev SV, Sedlak D, Baker TS, Strauss JH, Kuhn RJ, Rossmann MG. 2003. Structures of immature flavivirus particles. EMBO J 22:2604–2613. doi:10.1093/emboj/cdg270. 12773377 PMC156766

[8] Yu IM, Zhang W, Holdaway HA, Li L, Kostyuchenko VA, Chipman PR, Kuhn RJ, Rossmann MG, Chen J. 2008. Structure of the immature dengue virus at low pH primes proteolytic maturation. Science 319:1834–1837. doi:10.1126/science.1153264. 18369148

[9] Li L, Lok SM, Yu IM, Zhang Y, Kuhn RJ, Chen J, Rossmann MG. 2008. The flavivirus precursor membrane-envelope protein complex: structure and maturation. Science 319:1830–1834. doi:10.1126/science.1153263. 18369147

[10] Zheng A, Yuan F, Kleinfelter LM, Kielian M. 2014. A toggle switch controls the low pH-triggered rearrangement and maturation of the dengue virus envelope proteins. Nat Commun 5:3877. doi:10.1038/ncomms4877. 24846574 PMC4063126

[11] Zheng A, Umashankar M, Kielian M. 2010. In vitro and in vivo studies identify important features of dengue virus pr-E protein interactions. PLoS Pathog 6:e1001157. doi:10.1371/journal.ppat.1001157. 20975939 PMC2958806

[12] Pokidysheva E, Zhang Y, Battisti AJ, Bator-Kelly CM, Chipman PR, Xiao C, Gregorio GG, Hendrickson WA, Kuhn RJ, Rossmann MG. 2006. Cryo-EM reconstruction of dengue virus in complex with the carbohydrate recognition domain of DC-SIGN. Cell 124:485–493. doi:10.1016/j.cell.2005.11.042. 16469696

[13] Miller JL, de Wet BJ, Martinez-Pomares L, Radcliffe CM, Dwek RA, Rudd PM, Gordon S. 2008. The mannose receptor mediates dengue virus infection of macrophages. PLoS Pathog 4:e17. doi:10.1371/journal.ppat.0040017. 18266465 PMC2233670

[14] Liu Y, Liu J, Pang X, Liu T, Ning Z, Cheng G. 2015. The roles of direct recognition by animal lectins in antiviral immunity and viral pathogenesis. Molecules 20:2272–2295. doi:10.3390/molecules20022272. 25642837 PMC6272511

[15] Modis Y, Ogata S, Clements D, Harrison SC. 2004. Structure of the dengue virus envelope protein after membrane fusion. Nature 427:313–319. doi:10.1038/nature02165. 14737159

[16] Bressanelli S, Stiasny K, Allison SL, Stura EA, Duquerroy S, Lescar J, Heinz FX, Rey FA. 2004. Structure of a flavivirus envelope glycoprotein in its low-pH-induced membrane fusion conformation. EMBO J 23:728–738. doi:10.1038/sj.emboj.7600064. 14963486 PMC380989

[17] Zhang X, Ge P, Yu X, Brannan JM, Bi G, Zhang Q, Schein S, Zhou ZH. 2013. Cryo-EM structure of the mature dengue virus at 3.5-A resolution. Nat Struct Mol Biol 20:105–110. doi:10.1038/nsmb.2463. 23241927 PMC3593067

[18] Kostyuchenko VA, Zhang Q, Tan JL, Ng TS, Lok SM. 2013. Immature and mature dengue serotype 1 virus structures provide insight into the maturation process. J Virol 87:7700–7707. doi:10.1128/JVI.00197-13. 23637416 PMC3700294

[19] Parrish CR, Holmes EC, Morens DM, Park EC, Burke DS, Calisher CH, Laughlin CA, Saif LJ, Daszak P. 2008. Cross-species virus transmission and the emergence of new epidemic diseases. Microbiol Mol Biol Rev 72:457–470. doi:10.1128/MMBR.00004-08. 18772285 PMC2546865

[20] Li W, Wong SK, Li F, Kuhn JH, Huang IC, Choe H, Farzan M. 2006. Animal origins of the severe acute respiratory syndrome coronavirus: insight from ACE2-S-protein interactions. J Virol 80:4211–4219. doi:10.1128/JVI.80.9.4211-4219.2006. 16611880 PMC1472041

[21] Musso D, Gubler DJ. 2016. Zika virus. Clin Microbiol Rev 29:487–524. doi:10.1128/CMR.00072-15. 27029595 PMC4861986

[22] Lindquist L, Vapalahti O. 2008. Tick-borne encephalitis. Lancet 371:1861–1871. doi:10.1016/S0140-6736(08)60800-4. 18514730

[23] Zhang Y, Liang D, Yuan F, Yan Y, Wang Z, Liu P, Yu Q, Zhang X, Wang X, Zheng A. 2022. Replication is the key barrier during the dual-host adaptation of mosquito-borne flaviviruses. Proc Natl Acad Sci USA 119:e2110491119. doi:10.1073/pnas.2110491119. 35294288 PMC8944775

[24] Ansarah-Sobrinho C, Nelson S, Jost CA, Whitehead SS, Pierson TC. 2008. Temperature-dependent production of pseudoinfectious dengue reporter virus particles by complementation. Virology 381:67–74. doi:10.1016/j.virol.2008.08.021. 18801552 PMC3428711

[25] Dowd KA, Jost CA, Durbin AP, Whitehead SS, Pierson TC. 2011. A dynamic landscape for antibody binding modulates antibody-mediated neutralization of West Nile virus. PLoS Pathog 7:e1002111. doi:10.1371/journal.ppat.1002111. 21738473 PMC3128118

[26] Junglen S, Korries M, Grasse W, Wieseler J, Kopp A, Hermanns K, León-Juárez M, Drosten C, Kümmerera BM. 2017. Host range restriction of insect-specific flaviviruses occurs at several levels of the viral life cycle. mSphere 2:e00375-16. doi:10.1128/mSphere.00375-16. PMC5227070 28101536

[27] Wen D, Li S, Dong F, Zhang Y, Lin Y, Wang J, Zou Z, Zheng A. 2018. N-glycosylation of viral E protein is the determinant for vector midgut invasion by flaviviruses. mBio 9:e00046-18. doi:10.1128/mBio.00046-18. 29463651 PMC5821097

[28] Atieh T, Nougairede A, Klitting R, Aubry F, Failloux AB, de Lamballerie X, Priet S. 2017. New reverse genetics and transfection methods to rescue arboviruses in mosquito cells. Sci Rep 7:13983. doi:10.1038/s41598-017-14522-6. 29070887 PMC5656662

[29] Wang HJ, Li XF, Ye Q, Li SH, Deng YQ, Zhao H, Xu YP, Ma J, Qin ED, Qin CF. 2014. Recombinant chimeric Japanese encephalitis virus/tick-borne encephalitis virus is attenuated and protective in mice. Vaccine 32:949–956. doi:10.1016/j.vaccine.2013.12.050. 24394443

[30] Engel AR, Mitzel DN, Hanson CT, Wolfinbarger JB, Bloom ME, Pletnev AG. 2011. Chimeric tick-borne encephalitis/dengue virus is attenuated in Ixodes scapularis ticks and Aedes aegypti mosquitoes. Vector Borne Zoonotic Dis 11:665–674. doi:10.1089/vbz.2010.0179. 21142950 PMC3115420

[31] Pletnev AG, Men R. 1998. Attenuation of the Langat tick-borne flavivirus by chimerization with mosquito-borne flavivirus dengue type 4. Proc Natl Acad Sci USA 95:1746–1751. doi:10.1073/pnas.95.4.1746. 9465088 PMC19176

[32] Orlinger KK, Hofmeister Y, Fritz R, Holzer GW, Falkner FG, Unger B, Loew-Baselli A, Poellabauer EM, Ehrlich HJ, Barrett PN, Kreil TR. 2011. A tick-borne encephalitis virus vaccine based on the European prototype strain induces broadly reactive cross-neutralizing antibodies in humans. J Infect Dis 203:1556–1564. doi:10.1093/infdis/jir122. 21592984

[33] Zhang R, Kim AS, Fox JM, Nair S, Basore K, Klimstra WB, Rimkunas R, Fong RH, Lin H, Poddar S, Crowe JE, Doranz BJ, Fremont DH, Diamond MS. 2018. Mxra8 is a receptor for multiple arthritogenic alphaviruses. Nature 557:570–574. doi:10.1038/s41586-018-0121-3. 29769725 PMC5970976

[34] Ma H, Kim AS, Kafai NM, Earnest JT, Shah AP, Case JB, Basore K, Gilliland TC, Sun C, Nelson CA, Thackray LB, Klimstra WB, Fremont DH, Diamond MS. 2020. LDLRAD3 is a receptor for Venezuelan equine encephalitis virus. Nature 588:308–314. doi:10.1038/s41586-020-2915-3. 33208938 PMC7769003

[35] Clark LE, Clark SA, Lin C, Liu J, Coscia A, Nabel KG, Yang P, Neel DV, Lee H, Brusic V, Stryapunina I, Plante KS, Ahmed AA, Catteruccia F, Young-Pearse TL, Chiu IM, Llopis PM, Weaver SC, Abraham J. 2022. VLDLR and ApoER2 are receptors for multiple alphaviruses. Nature 602:475–480. doi:10.1038/s41586-021-04326-0. 34929721 PMC8808280

[36] Woolhouse M, Scott F, Hudson Z, Howey R, Chase-Topping M. 2012. Human viruses: discovery and emergence. Philos Trans R Soc Lond B Biol Sci 367:2864–2871. doi:10.1098/rstb.2011.0354. 22966141 PMC3427559

[37] Munderloh UG, Liu Y, Wang M, Chen C, Kurtti TJ. 1994. Establishment, maintenance and description of cell lines from the tick Ixodes scapularis. J Parasitol 80:533–543. doi:10.2307/3283188. 8064520

[38] Crill WD, Chang GJ. 2004. Localization and characterization of flavivirus envelope glycoprotein cross-reactive epitopes. J Virol 78:13975–13986. doi:10.1128/JVI.78.24.13975-13986.2004. 15564505 PMC533943

[39] Stiasny K, Kiermayr S, Holzmann H, Heinz FX. 2006. Cryptic properties of a cluster of dominant flavivirus cross-reactive antigenic sites. J Virol 80:9557–9568. doi:10.1128/JVI.00080-06. 16973559 PMC1617264

[40] Dai S, Zhang T, Zhang Y, Wang H, Deng F. 2018. Zika virus baculovirus-expressed virus-like particles induce neutralizing antibodies in mice. Virol Sin 33:213–226. doi:10.1007/s12250-018-0030-5. 29774519 PMC6013542

[41] Kumar S, Stecher G, Tamura K. 2016. MEGA7: Molecular Evolutionary Genetics Analysis Version 7.0 for bigger datasets. Mol Biol Evol 33:1870–1874. doi:10.1093/molbev/msw054. 27004904 PMC8210823

[42] Felsenstein J. 1985. Confidence limits on phylogenies: an approach using the bootstrap. Evolution 39:783–791. doi:10.1111/j.1558-5646.1985.tb00420.x. 28561359

